# Epicardial EMT and cardiac repair: an update

**DOI:** 10.1186/s13287-024-03823-z

**Published:** 2024-07-19

**Authors:** Eleonora Foglio, Erica D’Avorio, Riccardo Nieri, Matteo Antonio Russo, Federica Limana

**Affiliations:** 1 Technoscience, Parco Scientifico e Tecnologico Pontino, Latina, Italy; 2 Dipartimento di Promozione delle Scienze Umane e della Qualità della Vita, San Raffaele University of Rome, Rome, Italy; 3 Department of Experimental Medicine, Sapienza University of Rome, Rome, Italy; 4 IRCCS San Raffaele Roma, Rome, Italy; 5 Laboratorio di Patologia Cellulare e Molecolare, IRCCS San Raffaele Roma, Rome, Italy

**Keywords:** EMT, Epicardial cells, Cardiac repair and regeneration, Molecular rehabilitation

## Abstract

Epicardial epithelial-to-mesenchymal transition (EMT) plays a pivotal role in both heart development and injury response and involves dynamic cellular changes that are essential for cardiogenesis and myocardial repair. Specifically, epicardial EMT is a crucial process in which epicardial cells lose polarity, migrate into the myocardium, and differentiate into various cardiac cell types during development and repair. Importantly, following EMT, the epicardium becomes a source of paracrine factors that support cardiac growth at the last stages of cardiogenesis and contribute to cardiac remodeling after injury. As such, EMT seems to represent a fundamental step in cardiac repair. Nevertheless, endogenous EMT alone is insufficient to stimulate adequate repair. Redirecting and amplifying epicardial EMT pathways offers promising avenues for the development of innovative therapeutic strategies and treatment approaches for heart disease. In this review, we present a synthesis of recent literature highlighting the significance of epicardial EMT reactivation in adult heart disease patients.

## Introduction

Over the last decade, several strategies have been applied to promote cardiac repair, including stimulation of cardiomyocyte cell cycle re-entry, treatment with stem/progenitor cells or stem cell-derived cardiomyocytes, modulation of tissue repair using cytokines and growth factors, and tissue engineering approaches [1, 2]. An improved understanding of the molecular mechanisms driving cardiomyocyte (CM) proliferation and differentiation and, importantly, the role of noncardiomyocytes in supporting cardiac function is critical for achieving significant cardiac regeneration.

Processes occurring during cardiac development are often recapitulated in the disease setting. For this reason, many efforts have been devoted to the development of new strategies capable of modulating these processes to bolster tissue repair. The epithelial-to-mesenchymal transition (EMT) is a fine representation of this phenomenon. Specifically, recent findings support the involvement of epicardial EMT in cardiac repair and regeneration by providing progenitor cells with the ability to differentiate into different cardiac cell phenotypes and secrete paracrine factors [3–5].

Epicardial EMT consists of the migration of epicardial cells from the surrounding monolayer to generate subepicardial mesenchyme, which eventually differentiates into cells of the mesenchymal lineage.

During cardiac development, epicardial cells undergo EMT and migrate into the subepicardial space, giving rise to EPDCs. These EPDCs then invade the myocardial wall, where they differentiate into different phenotypes of the developing heart, including coronary endothelial cells, smooth muscle cells and cardiac fibroblasts [6–8]. Furthermore, epicardial cells provide soluble factors that stimulate coronary vessel development along with cardiomyocyte proliferation and differentiation [3, 9–11].

In the adult heart, following injury, epicardial cells initiate an embryonic-like response, and EMT represents the first critical step involved in epicardial-mediated cardiac repair and regeneration [5, 11].

In this review, we will focus on the latest updates concerning EMT as a pivotal process for heart growth and repair.

## Epithelial-mesenchymal transition (EMT): an overview

Epithelial-mesenchymal transition (EMT) is a process through which epithelial cells gradually lose their epithelial features, such as intracellular adhesion and apical-basal polarity, and acquire mesenchymal-like cell properties, such as migratory and invasive capacity, enhanced resistance to apoptosis and increased capability to produce ECM components [12, 13]. Conversely, the reverse process, mesenchymal–epithelial transition (MET), allows the generation of epithelial cells from mesenchymal cells [14].

Three distinct types of EMTs have emerged from the literature, each involved in a specific biological contest: (1) Type-1 EMT is involved in embryogenesis and subsequent development stages, such as embryo implantation, gastrulation, and neural crest formation [12, 15], ultimately contributing, together with MET, to the formation of a fully functional embryo [12]; and (2) Type-2 EMT is activated in response to inflammation and injury, playing a role in repair and regeneration processes [14, 16, 17]. Moreover, it is linked to fibrosis due to its promotion of myofibroblast proliferation, potentially leading to organ dysfunction. [18]. Finally, (3) Type 3 EMT is predominantly observed in cancer, mostly during metastasis, allowing tumor cells to acquire a mesenchymal phenotype that enhances their invasiveness, motility, and apoptosis resistance [19, 20].

Concerning the molecular aspect of EMT, cells connect through different types of junctions, such as tight junctions and adherent junctions, which are important for maintaining epithelial integrity [21, 22]. The most important events in EMT are the destabilization and disintegration of cell junctions and the loss of epithelial cell markers.

Specifically, E-cadherin, a transmembrane adhesion protein, is downregulated due to degradation, while vimentin is upregulated, resulting in reduced transport of E-cadherin to the cell surface [23]. The reduced expression of proteins such as claudin and ZO-1 (zonula occludens 1), as well as connexins, leads to the impairment of tight and gap junctions, while the alteration of cytoskeletal proteins, rearrangement of actin structure and upregulation of integrins contribute to the formation of lamellipodia and filopodia to increase cell motility [24, 25]. In addition, several genes encoding mesenchymal markers, such as vimentin, fibronectin, neural cadherin (N-cadherin) and matrix metalloproteases (MMPs), are upregulated by different transcription factors (TFs), including SNAI1/Snail, SNAI2/Slug, Zeb1/2, LEF-1, and Twist, which function as repressors of E-cadherin by interacting with its promoter region [13, 26, 27]. These TFs are subsequently triggered by different signaling molecules and pathways, such as components of the transforming growth factor β (TGF-β), hepatocyte growth factor (HGF), insulin growth factor (IGF), epidermal growth factor (EGF), platelet-derived growth factor (PDGF), insulin growth factor-1 (IGF-1), Wnt, Notch, hypoxia, and receptor tyrosine kinase (RTK) pathways [12, 28]. Notably, noncoding miRNAs can influence TF expression during EMT; for instance, the downregulation of miR-200, miR-34a or miR-137 promotes EMT progression by upregulating the expression of several TFs, such as Zeb1/Zeb2 [29].

TGF-β ligands bind to many receptors (TGF-βRI, TGF-βRII, and TGF-βRIII), leading to SMAD2/3, PIK3 (phosphatdyl-inositol-3-kinase) and RAS pathway activation to increase EMT-related TF expression. Although TGF-β members are strong inducers of EMT during development, several studies have reported their involvement in EMT during pathological conditions such as cancer, metastasis, and fibrosis. For example, TGF-β1, together with the Notch, Wnt and MAPK pathways, can induce EMT in tumor cells, which in turn moves from the primary tumor site to other sites and leads to invasion and metastasis. Additionally, TGF-β-induced EMT appears to be crucial for the induction and maintenance of cancer stem cells; in particular, an increase in CD44high/CD24low breast cancer stem cells, recognized for their high tumorigenic potential [30, 31], together with significant cell migration and invasion in lung adenocarcinoma [32] and an increased probability of mesenchymal phenotype acquisition in hepatocarcinoma cells [33], was noted.

Many studies have suggested that both EMT and MET play key roles not only during development but also in the repair of adult organs and tissues [17, 34]. These processes are implicated in both physiological and pathological events such as embryogenesis, wound healing, tumor growth and fibrosis [35, 36].

For instance, EMT, alongside its inducer TGF-β, plays a critical role in the process of cutaneous wound healing, where keratinocytes move across the wound to support re-epithelialization and recovery via SMAD-dependent or SMAD-independent pathways, promoting mesenchymal marker expression [36–39]. There is further evidence indicating that EMT is involved in the repair of extracutaneous organs, such as the heart, after myocardial injury [40, 41].

During physiological repair, tissue integrity is restored through the formation of a resilient scar facilitated by myofibroblasts. Nevertheless, the prolonged pathological activity of myofibroblasts, which primarily originate from EMT, may lead to the onset of fibrosis [36, 42]. Indeed, elevated levels of TGF-β have been found in the kidney [43], liver [44], lung [45], and heart under fibrotic conditions, revealing the crucial role of TGF-β in processes such as endothelial-to-mesenchymal transition (EndMT) [46].

The influence of factors such as the microenvironment and the duration of injury on the balance between EMT-driven fibrosis and regeneration is apparent. These factors determine which process predominates, with one potentially overriding the other [47].

It is important to note that both EMT and MET are not discrete events but rather exist as transitional states. Cells exhibit a dynamic interplay of partial EMT, MET, and various EMT stages to ensure the proper completion of organ development [12], as observed in cell types such as endocardial cells, notochords, splanchnopleura, and somatopleura [17, 48]. This transitional nature underscores the malleability and reversibility inherent in these processes.

Given these complexities, further studies are necessary to identify molecules or compounds that can target the EMT process to mitigate cancer cell proliferation, metastasis, and fibrosis. The ultimate goal is to enhance the sensitivity of tumor cells to therapeutic interventions or to stimulate tissue regeneration to overcome fibrosis. However, the effectiveness of different strategies against EMT—whether through targeting EMT factors, inducers, or cell-specific molecules [49–51]—is hampered by the presence of transient and incomplete EMT states. Therefore, a better understanding of this process and its inhibitors is essential.

## Epicardial EMT during cardiac development

**Fig. 1:**
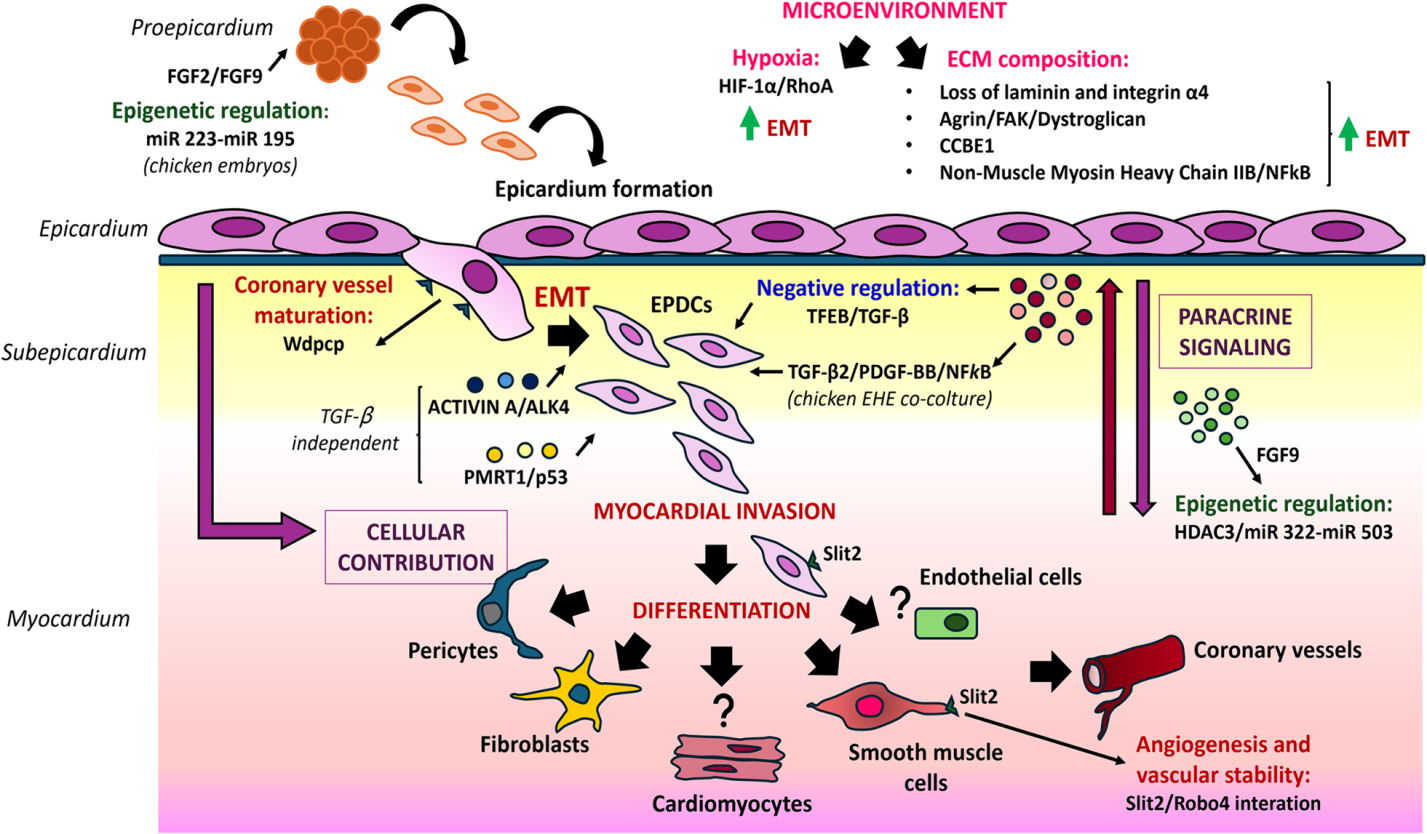
Epicardial EMT during cardiac development. Epicardial EMT during cardiac development is a tightly controlled process involving various myocardial and epicardial-derived signals. In recent years, novel signaling pathways (either TGF-β dependent or independent) and epigenetic regulators have emerged as involved in epicardial–myocardial reciprocal communication during heart development, influencing epicardial EMT and, consequently, myocardial expansion and compaction, as well as maturation and remodeling of the primitive coronary plexus. Moreover, recently, scientific research has focused on elucidating the critical features of the cardiac microenvironment that influence epicardial EMT during embryonic development. Specifically, hypoxia and the proper composition of the extracellular matrix (ECM) are fundamental for orchestrating tight and dynamic spatiotemporal regulation of heart development

During epicardial formation (at approximately E12.5 in mice), a subset of epicardial cells undergo EMT, acquiring mesenchymal and migratory features; these cells detach from the epicardial layer, degrade the underlying basal membrane and migrate into the subepicardial space. In addition to these cellular contributions, the epicardium and EPDCs produce paracrine signaling factors critical for crosstalk with other compartments of the developing myocardial wall (e.g., myocardium and coronary endothelial cells) [3, 52]: loss of communication causes myocardial hypoplasia and abnormal coronary vascular development [53, 54]. Here, we summarize the latest findings on epicardial EMT during cardiac development (Fig. 1).

### New EMT signaling pathways involved in epicardial-myocardial crosstalk

Epicardial EMT during cardiac development is a tightly controlled process involving various myocardial and epicardial-derived signals that act in a paracrine or autocrine manner, respectively, and play crucial roles as regulators of epicardial EMT [47, 55].

Growth factors originating from both the epicardium and the myocardium are well known to be involved in epicardial–myocardial reciprocal communication during heart development, influencing epicardial EMT and, consequently, myocardial expansion and compaction. The role of bone morphogenic proteins (BMPs) and fibroblast growth factors (FGFs) has been reported early during the formation of the proepicardium/septum transversum (PE/ST) [56], where they direct the pericardial mesoderm to either a proepicardial or myocardial fate [57, 58]. Specifically, Dueñas et al. demonstrated that BMP2 and BMP4 promote cardiomyocyte formation, while FGF2 and FGF8 induce epicardial differentiation in chicken embryos via the regulatory roles of miR-223 and miR-195 [59]. In a subsequent study, following the administration of different BMP and FGF family members to mouse PE/ST explants [60], they surprisingly found that neither the FGF2/FGF8-induced modulation of EMT markers nor the miR-223/miR-195 inductive potential found in chicken embryos were conserved in mice, highlighting species-specific variations in PE/ST development [61]. Further RNA-seq analyses in mice revealed differential expression of additional BMP and FGF family members during PE development, suggesting their potential roles in this process. In another recent study, epigenetic regulation of epicardial EMT through dual inhibitory control by HDACs and miRs of FGF growth factors released by the epicardium and EPDCs during heart formation was demonstrated [62]. Specifically, deletion of histone deacetylase 3 (HDAC3) in the epicardium led to reduced EPDC derivation and invasion of the compact myocardium. In both in vitro and in vivo* HDAC3-deficient epicardial cells*, miR-322 and miR-503 were significantly upregulated, whereas FGF9 expression was suppressed, leading to decreased CM proliferation. The authors speculated that during development, the epicardium induces ventricular myocardial wall expansion through paracrine signaling, i.e., stimulating FGF9 expression by repressing miR-322/miR503 through HDAC3 deacetylase activity.

The TGF-β pathway is the most extensively studied signaling pathway involved in the induction of epicardial EMT and subsequent differentiation of the EPDC lineage [63, 64]. TGF-β family ligands (e.g., TGF-β1/2/3) induce intracellular signaling responses upon activation of a heterodimeric transmembrane receptor complex consisting of Type I (ALK5) and Type II receptors (TGF-βRII) with enzymatic serine-threonine kinase activity [65]. Activation of this enzymatic complex leads to the phosphorylation of SMAD2/SMAD3, which then forms heterodimeric nuclear complexes with SMAD4 to modulate the expression of a specific subset of genes encoding TFs critical for EMT [66].

A proteomic approach involving two-dimensional liquid chromatography-mass spectrometry/mass spectrometry (2D-LC‒MS/MS) combined with in-depth bioinformatics analysis was adopted by Li and coauthors to identify novel factors released into the medium by chicken EPDC-heart explant (EHE) coculture, a model that preserves the crosstalk between the epicardium and myocardium [67]. Through this approach, they generated an EHE secretome dataset of proteins directed toward the extracellular space or cell membranes, and by the use of bioinformatic tools, they predicted regulatory networks implicated in epicardial-myocardial signaling. The EHE dataset highlighted NF-κB as a pivotal signaling hub with many known targets of NF-κB signaling found in the EHE secretome. The authors performed cell-based functional assays to validate this result and demonstrated that NF-κB activation is necessary for the transition of chicken and mouse epicardial cells from an epithelial to mesenchymal state in response to TGF-β2/PDGF-BB treatment.

A recent investigation demonstrated that the oncogenic transcription factor EB (TFEB) functions as a negative regulator of both TGF-β-driven epicardial EMT and EPDC differentiation and invasion during heart development [68]. In a Tfeb-EGFP-expressing mouse model, Tfeb expression, which was downregulated during EMT, was first observed in Wt1^+^ epicardial cells, specifically upon activation of TGF-β1 signaling. Conversely, a mouse model with sustained Tfeb expression in EPDCs exhibited embryonic lethality, possibly due to the inhibitory effect of Tfeb overexpression on the EMT-mediated differentiation of epicardial cells into fibroblasts and vascular SMCs. Experiments in primary epicardial cells and a mouse embryonic epicardial cell line (MEC) confirmed that elevated TFEB levels specifically impede TGF-β-induced EMT, while Tfeb-silenced cells are prone to EMT without any additional triggers and present enhanced sensitivity to low doses of TGF-β1. Notably, TFEB mediates the cell response to TGF-β1 by upregulating the expression of thymine-guanine-interacting factor 1 (TGIF1), a suppressor of TGF-β-induced SMAD-mediated transcription [69]. TFEB activates the Tgif1 promoter, leading to increased expression of TGIF1 in association with Tfeb overexpression, as shown by in vitro and in vivo findings.

Recently, new signaling pathways that act upstream or independently of TGF-β have been implicated in the regulation of epicardial EMT.

The role of Activin A and its receptor ALK4 in inducing EMT in multiple cancer cell lines has been extensively explored [70–73]. The presence of activin A in subepicardial tissue has already been reported [74], and more recently, a single-cell RNA sequencing (scRNA-seq) dataset of the embryonic mouse epicardium revealed ALK4 mRNA expression in a subset of epicardial cells [75]. Dronkers and colleagues demonstrated the ability of activin A to regulate epicardial EMT through the activation of its receptor ALK4 [76]. They established a cell culture model for human primary adult and fetal epicardial EMT, allowing simultaneous evaluation of inhibitors and enhancers of specific pathways involved in the regulation of this process [77]. Exogenous Activin A stimulation of adult epicardial cells led to a clear acquisition of mesenchymal behavior in these cells; conversely, fetal epicardial cells, which are capable of spontaneously undergoing EMT, preserved their epithelial phenotype when exposed to the natural antagonist of Activin known as Follistatin (FST). The regulation of EMT by the activin A/ALK4 axis was TGF-β independent and potentially compensated for the inactivation of TGF-β signaling; notably, a synergistic effect was observed in blocking spontaneous EMT in fetal epicardial cells when TGF-β-capturing antibodies (to inhibit TGFβ signaling) and FST (to inhibit activin signaling) were concurrently administered compared to individual treatments.

An additional novel pathway regulating epicardial EMT during embryonic heart development without affecting TGF-β-induced SMAD3 signaling involves the PRMT1-p53 axis. PRMT1 is a protein responsible for 75% of arginine residue methylation on histones and nonhistone proteins in mammalian cells [78]. Using scRNA-seq, Jackson-Weaver et al. demonstrated that the PRMT1-p53 pathway is required for epicardial EMT and EPDC invasion and differentiation, leading to the formation of cardiac mesenchymal lineages. Specifically, they found that epicardial-specific deletion of PRMT1 causes significant dysregulation of alternative splicing mechanisms of Mdm4, a protein that interacts with p53 in the apoptotic process, which, in turn, stabilizes p53 and causes its accumulation and enhanced p53-mediated degradation of Slug, thereby inhibiting epicardial EMT [79].

The epicardium is also an important source of chemokines that regulate coronary vessel patterning. The maturation and remodeling of the primitive coronary plexus involve the integration of smooth muscle cells (coronary mural cells), derived from the epicardial sheet following EMT events, into the primitive endothelial network [80–82]. In this context, Liu and collaborators proposed a role for Wdpcp, a protein involved in actin cytoskeleton modulation and the regulation of directional cell movement [83, 84], in the process of coronary vessel maturation [85]. They illustrated that Wdpcp deficiency in the epicardium caused defects in the remodeling of the primitive plexus due to impaired EMT and EPDC migration. Lineage tracing experiments using epicardium-specific Wt1^CreERT2^ and Rosa26^mTmG^ reporters [86] revealed a reduced number of EPDCs due to defective EMT and impaired infiltration of these cells into the myocardial wall in a three-dimensional (3D) gel invasion assay. This observation was further supported by flow cytometric analysis of dissociated E14.5 heart ventricles lacking Wdpcp, which indicated a reduced number of EPDCs (GFP^+^) compared to that in wild-type hearts. Additionally, the expression of EMT markers (Snail2 and Twist1) and mesenchymal markers (vinculin and vimentin) was significantly reduced, indicating reduced EMT.

The importance of paracrine signaling provided by epicardial cells undergoing EMT to proper EC localization and fate specification in the developing heart [87] was recently highlighted by Quijada and collaborators. In a prior study, they revealed that genetic disruption of epicardial EMT in mice [88] led to alterations in the developmental trajectory of EC, with the accumulation of an immature EC population within the subepicardium. scRNA-seq of EPDCs and coronary ECs at critical developmental stages revealed the induction of Slit2 during epicardial EMT in a subset of epicardial-derived mural cells, which act as vascular “guidepost cells”. Slit2^+^ cells were identified near Robo4^+^ ECs in the subepicardium, thus confirming the significance of Slit2-Robo4 interactions in regulating angiogenesis and vascular stability, as described in other studies [89, 90]. Furthermore, the authors demonstrated that epicardial EMT, which drives the differentiation of EPDCs into vascular mural lineages [88], also induced the expression of specific chemotactic signals in distinct populations of mural cells (while concurrently silencing some mesothelial cues), providing precise positional information to control EC patterning.

### Microenvironmental conditions impacting epicardial EMT activation

The native heart microenvironment, which includes biochemical cues, mechanical stimulation, synchronized electrical networks and cell-cell/cell-matrix interactions, offers the complex and dynamic milieu required for heart development [91]. Recently, scientific research has focused on elucidating the critical features of the cardiac microenvironment that influence epicardial EMT during embryonic development.

#### Hypoxia

During heart development, different degrees of hypoxia exist in specific regions, with cellular adaptations to hypoxia predominantly mediated by hypoxia-inducible factor-1α (HIF-1α): depletion of HIF-1α in mice severely disrupts myocardial and vascular endothelial development, leading to embryonic death around E10 [92, 93].

HIF-1α also plays a complex regulatory role during specific stages of epicardial development: the epicardium has been identified as a hypoxic microenvironment suitable for harboring progenitor cells during embryogenesis and directing their differentiation [94, 95]. In a study by Tao and colleagues in 2013, some epicardial regions presented a greater abundance of HIF-1α-positive epicardial cells than other regions. These cells underwent EMT more readily, while their ability to migrate into the myocardium was restrained, thereby enabling their differentiation and incorporation into large vessels that originate from the epicardium. Conversely, regions with lower levels of HIF-1α in epicardial cells experience delayed EMT, allowing precursor cells to migrate considerable distances into the myocardium under the influence of VEGF signaling [96]. Furthermore, based on evidence that HIF-1α promoted the activation of Snail in Tbx18^+^ epicardial cells [97], as well as the expression of the EMT-related transcription factor Twist2 [96], Tao et al. demonstrated the contribution of microenvironmental hypoxia to enhancing epicardial EMT and promoting EPDC differentiation into vascular smooth muscle cells (SMCs) via a noncanonical TGF-β molecular pathway [98]. Specifically, this process is mediated through the activation of the RhoA pathway (a direct target of HIF) [99]; chemical and genetic approaches used to inhibit the downstream RhoA target Rho kinase (ROCK) decreased the expression of SMA and SM22 in EPDCs under hypoxic conditions.

#### Extracellular matrix (ECM)

The extracellular matrix (ECM) is a noncellular component of the cardiac microenvironment with essential structural and regulatory roles in providing the structural support and regulatory functions necessary for tissue architecture and cellular function [100]. In recent years, its importance in orchestrating tight and dynamic spatiotemporal regulation of epicardiogenesis has emerged [101]. Spatiotemporal scRNA-seq data from developing chicken hearts revealed a significant upregulation of different ECM factors implicated in cell migration during epicardial EMT [102].

Regions exhibiting active EMT are characterized by the presence of specific matrix components in the epicardial layer. For instance, laminin, a key component of the ECM-associated basement membrane (reviewed by Yurchenco, 2015) [103], was found to be reduced and discontinuous among epicardial cell clusters in regions of active EMT, suggesting the presence of a disrupted basement membrane that may facilitate EPC migration; likewise, integrin α4 was significantly downregulated in regions exhibiting heightened EMT [104].

Agrin is another important component of the ECM that ensures connectivity between cells and the basement membrane [105]. Its role in the embryonic heart, specifically in ensuring the proper deposition of epicardial ECM, has already been described [106]. Loss of agrin compromised several ECM components of the epicardium, indicating the comprehensive involvement of agrin in ECM structural organization. Mechanistically, agrin acts through its receptor dystroglycan to transmit tissue rigidity signals and connect extracellular cues from the ECM with intracellular pathways [107]. Sun et al. observed for the first time that agrin promoted epicardial EMT in both mouse and human embryonic models through a mechanism that involved the activation of the integrin-focal adhesion kinase (FAK) signaling cascade and the aggregation of dystroglycan in the Golgi apparatus, leading to the stabilization of YAP and activation of the Hippo–YAP pathway [104]. As a confirmation, the loss of agrin and the consequent disaggregation and dispersion of dystroglycan resulted in abnormal and reduced EMT in epicardial cells.

Collagen- and calcium-binding EGF-like domain 1 (CCBE1) represents another essential ECM protein that has been recently implicated in proper coronary vasculature development, as highlighted by impaired coronary vascularization in Ccbe1 mutant mice (Ccbe1^tm1Lex^) [108, 109]. Bonet and his group discovered that CCBE1 loss of function also disrupted epicardial cell proliferation and EMT. Although CCbe1 mutants had no obvious alterations in the formation of epicardial layers, they had a diminished proliferation rate in epicardial cells compared to wild-type hearts at the E12.5, E13.5 and E14.5 stages, significantly impacting myocardial growth [110]. Moreover, in vitro assays indicated reduced epicardial migration in Ccbe1^tm1Lex^ epicardial explants, suggesting EMT impairment. This observation was corroborated by transcriptome analysis of the mRNA of ventricles from Ccbe1^tm1Lex^ hearts, which showed dysregulation of EMT-related genes.

Nonmuscle myosin heavy chain IIB, a key component of the actin-myosin cytoskeletal machinery, is important for cell migration [111, 112]. Loss of function of this protein in mice (known as *Myh10Δ* mutation) caused coronary vessel abnormalities, alterations in epicardial morphology (associated with impaired epicardial EMT and reduced migration of EPDCs into the myocardium) and perturbation of subepicardial ECM composition [113]. The subepicardial ECM plays an important role in epicardial function, specifically in the adhesion of the epicardial monolayer to the myocardium and in facilitating epicardial-myocardial molecular communication [52, 114]. Surprisingly, NMHC IIB-null epicardial cell culture does not exhibit any defects in vitro or appropriate EMT activation [115]. A key difference between the in vitro system and the mutant embryos is represented by the absence of the extracellular environment. The results by Ridge et al. indicated impaired NF-κB pathway activation in *Myh10Δ* mutants, suggesting that alterations in the subepicardial ECM of mutant embryos could hinder TGF-β and PDGF signaling upstream of the NF-κB pathway, thus contributing to epicardial EMT dysregulation.

### New methods to track cellular heterogeneity in the developing epicardium

An ongoing debate revolves around whether heterogeneous epicardial subpopulations already exist within the PEO or whether they are multipotent progenitors that become specific only after EMT [116]. Cre-based fate mapping analyses have long been the preferred approach for epicardial cell identification based on the assessment of several *bona fide* epicardial transcription factors, such as Wt1, Tbx18 and Tcf21 [7, 117, 118]. However, the expression patterns of these factors are neither exclusive to the epicardium nor uniformly expressed in the epicardial/subepicardial mesenchyme, thus rendering them inadequate for conducting lineage tracing experiments [119].

#### Epicardial subpopulations during development

Recently, scRNA-seq and multiplexed single-molecule RNA in situ hybridization (RNAscope) have been used to investigate epicardial heterogeneity and function across various organisms, such as zebrafish, chickens, mice and humans, and have yielded contrasting results [3, 75, 102, 116].

Weinberger and colleagues characterized the transcriptome of the developing zebrafish epicardium at the single-cell level [116]. They identified and functionally characterized three different epicardial cell subpopulations (named Epi1, Epi2, and Epi3), each characterized by a unique transcriptomic profile and spatial distribution within the developing heart. Among them, only the Epi1 population harbored cells coexpressing the *bona fide* epicardial signature genes Tcf21, Tbx18, and Wt1 [117, 120]. Functional perturbation studies and GO term analyses of genes prominently expressed in Epi1 indicated a possible role for this subpopulation in the formation of the epicardial cell sheet that migrates to envelope the myocardium. In contrast, the other subpopulations were not involved in the maintenance of epicardial integrity but in the formation of the outflow tract (Epi2) and the recruitment of myeloid cells to the developing heart (Epi3).

Mantri et al. combined single-cell and spatial transcriptomics (high-throughput scRNA-seq and spatially resolved RNA-seq) to analyze the transcriptional profiles of epicardial cells and EPDCs across different stages of ventricular development in chickens. According to previous studies [75, 121], EPDCs undergo EMT into the myocardium before fate determination since they maintain a progenitor-like transcriptional profile from the early to late four-chambered heart stages [102].

Using scRNA-seq analysis, Lupu et al. provided evidence that in mice, EPCD fate is determined only after EMT in response to environmental stimuli, and importantly, the marker expression profile of these cells before EMT does not constrain cell fate choice [75]. This model contradicted an earlier notion regarding the existence of different epicardial subpopulations with predetermined cell fates [122]. To explain this gap, Lupu et al. emphasized the necessity of distinguishing epicardial cells (placed on the surface of the heart) from EPDCs (in the subepicardial mesenchyme or within the myocardium) to obtain reliable results, suggesting that previous studies reporting heterogeneity did not investigate the PEO or the earliest stages of epicardial formation but only the epicardium after initiation of EMT.

In a very recent study, using an established protocol to separate the human epicardium from the underlying cardiac tissue [77], Streef et al. generated an epicardial cell-enriched dataset containing both epithelial-like epicardial cells and their mesenchymal derivatives. By analyzing the composition and function of the human fetal epicardium using this dataset, they overcame the limitations of using conventional markers [3]. Their scRNA-seq analysis revealed two closely related populations of epithelial cells, showing no functional difference between these populations and, therefore, again suggesting heterogeneity in the epicardium, potentially attributed to ongoing EMT or differentiation. Furthermore, the dataset also revealed several novel markers for better identification of epicardial cells in both the epithelial (e.g., CRIP1) and mesenchymal (e.g., NRK) stages. CRIP1 was previously suggested to be a positive regulator of epicardial EMT [88, 123], and the authors found that knockdown of CRIP1 rapidly induced EMT and migration in epicardial cell culture, suggesting that CRIP1 functions as an essential co-factor in maintaining epithelial homeostasis.

Another open issue concerns the origin of coronary endothelial cells (CECs) during development, raising questions about the vasculogenic potential of epicardial cells. Cre-loxP genetic lineage tracing identified the sinus venosus and ventricular endocardium as primary contributors [124–127]. However, a subset of murine proepicardial cells expressing the transcription factor Scleraxis (Scx) and the chemokine semaphorin 3D (Sema3D), distinct from the established PEO markers Wt1 and Tbx18, was proposed to give rise to the endocardium and coronary endothelium [128]. Conversely, Lupu et al. revealed the coexpression of all canonical epicardial markers (Wt1, Tcf21, Tbx18) together with Sema3d and Scx in the PEO and in the entire epicardial layer early in development, refuting the existence of subcompartments that might contribute to the coronary endothelium via the epicardial layer [75]. This discrepancy from a previous report [128] may reflect a failure to properly distinguish PEO from the septum trasversus mesenchyme (STM). Lupu et al. proposed Upk3b as a more selective PEO marker that could be used to delineate the boundary between PEO and other progenitors in the STM region.

#### Human-induced (hi)PSC-derived epicardial-like (EPI) cells and epicardioids

The unavailability of human embryonic tissue at the early stages of epicardial development has resulted in substantial gaps in the knowledge of human epicardial development and function.

The use of human-induced (hi)PSC-derived epicardial-like (EPI) cells to recapitulate epicardiogenesis in vitro is a recent advancement introduced to investigate epicardial development. Using these cells, Junghof et al. focused their work on the epicardial cell surfaceome, which encompasses all surface and transmembrane proteins crucial for the expansion, migration, and invasion of the epicardium during heart development [129]. They identified the type II classical cadherin CDH18 as a specific surface biomarker exclusively expressed in the fetal-stage epicardium in correlation with Wt1 and Tcf21 and hypothesized that CDH18 plays a pivotal role in the specification and maintenance of epicardial cell identity [130]. Accordingly, loss of CDH18 expression led to decreased cell proliferation, enhanced stabilization and increased β-catenin levels in hiPSC-derived EPI cells, thus activating downstream signaling pathways that drive cell fate toward SMC differentiation. RNA-Seq analysis confirmed SMC differentiation and revealed that TGF-

β signaling pathway activation upon decreased CDH18 expression.

Recently, Meier et al. developed human pluripotent stem cell-derived cardiac organoids termed epicardioids, which exhibit retinoic acid (RA)-dependent self-organization and functional ventricular myocardium with an external epicardial layer [131]. Remarkably, epicardioids could represent a valuable tool for investigating epicardial development, as they can dynamically replicate the sequential stages of epicardial and myocardial development and maturation [commented in [132]]. Consequently, time-course single-cell genomic analysis in epicardioids combined with lineage tracing could become useful for revealing the developmental pathways of the epicardial lineage and functional crosstalk with other cardiac cell types.

Intriguingly, using epicardioids, Meyer et al. attempted to address open questions related to epicardial heterogeneity and fate potential. Importantly, by linearly tracing CDH1^+^ mesothelial epicardial cells, the authors supported, at least in vitro, the hypothesis of the myocytic potential of the embryonic epicardium at early stages*.* Moreover, through the examination of chromatin accessibility patterns in all three epicardial derivatives (fibroblasts, SMCs and CMs), they corroborated the findings of earlier studies [3, 75, 102], indicating that fate determinations or epicardial cells occur after epicardial EMT and are not predetermined in distinct epicardial subpopulations [131].

## Epicardial EMT after myocardial injury

Epicardial EMT also serves as a crucial intermediate stage in the adult heart during regeneration since it converts the epicardium into a coordinator of cellular and paracrine responses that trigger cardiac repair following injury [5].

**Fig. 2:**
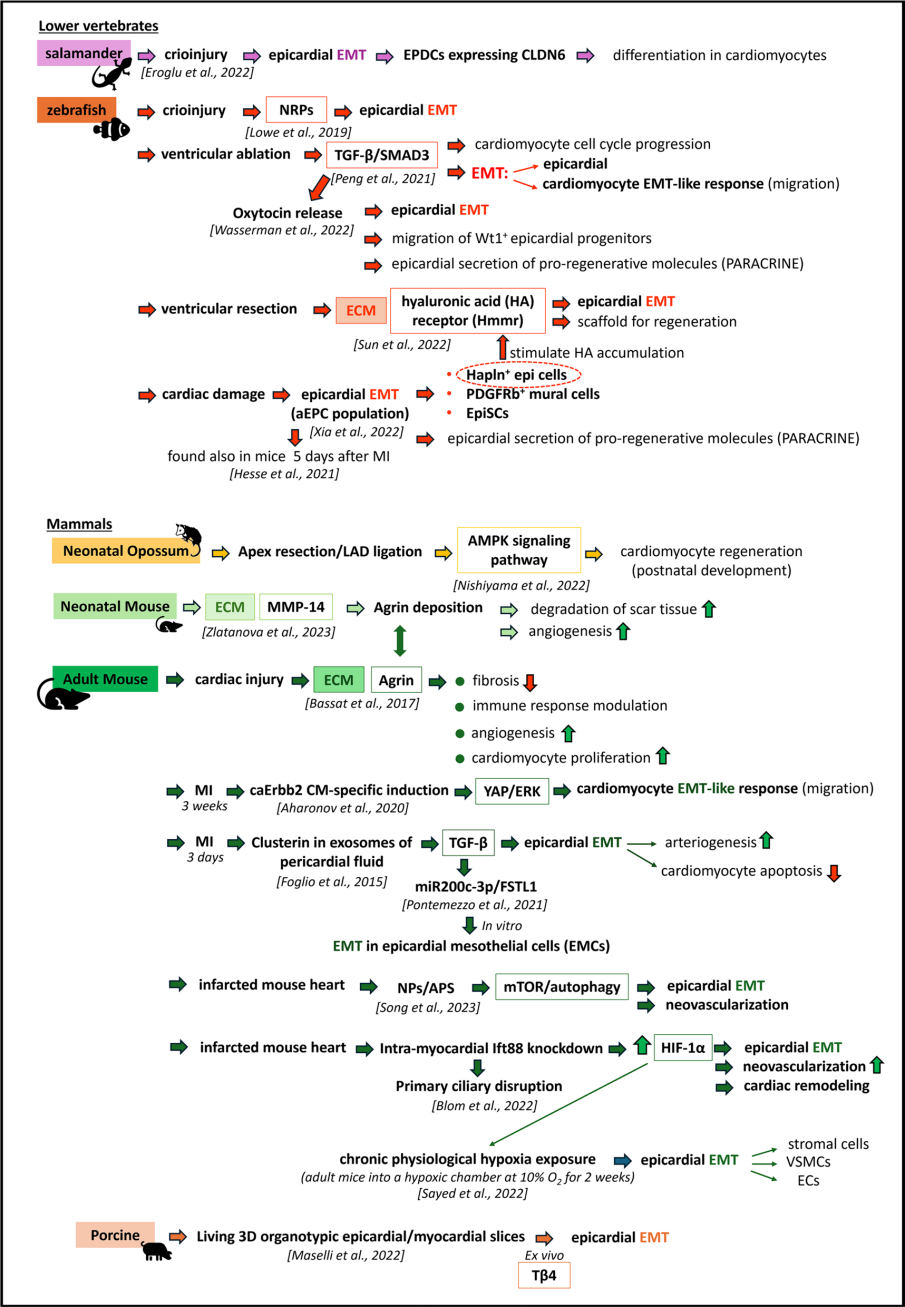
Epicardial EMT-mediated cardiac repair across vertebrate species. Schematic representation of the influence of new signaling pathways and of the role of extracellular matrix molecules in activating the adult epicardium via EMT leading to cardiac repair and regeneration in neonatal and adult hearts

A wide range of overlapping signaling pathways support EMT in the adult heart, and these pathways have already been extensively reviewed elsewhere [47, 133, 134]. In this part of the review, we will discuss the latest findings concerning the involvement of epicardial EMT in the context of heart repair (Fig. 2).

### Epicardial heterogeneity can give rise to species-specific differences during heart repair

It is well known that adult lower vertebrate, like teleost zebrafish and certain urodele amphibians, are renowned for their heightened capacity to regenerate damaged heart muscle [135, 136] achieved through the proliferation of existing CMs [137, 138]. Conversely, mammals lose this regenerative capacity shortly after birth, and after this period, injury results in scarring [139]. The mechanism behind this loss of regenerative capacity may not solely depend on species-specific intrinsic characteristics of CMs such as the limited ability of mature cardiomyocytes to reenter the cell cycle and proliferate to repair the ischemic tissue [140, 141].

Non-myocytes, including epicardial cells, are known to play critical roles in cardiac regeneration by influencing post-myocardial infarction adaptive immune regulation [142], extracellular matrix turnover, angiogenesis and cardiomyocyte growth [5], through both cellular (as a source of progenitor cells) and paracrine mechanisms [143]: for this reason, variation in regenerative potential among species could be attributed to factors that go beyond the proliferative capacity of cardiomyocytes.

An unresolved question in this context concerns whether the whole epicardial population can participate to the regenerative process or whether distinct cell types within the epicardial layer possess specific abilities in the reparative response [3]. Since epicardial cells have the potential to differentiate into different cardiac cell types, it has been hypothesized that the epicardial layer is not composed of one specific cell type. Independent studies recently revealed epicardial heterogeneity both in mouse Tβ4-reactivated epicardial-derived cells and zebrafish tcf21-expressing cells purified from uninjured adult hearts [144, 145]. More recently, fate mapping and trace lineage studies of epicardial cells both in embryonic and adult mice, chicks, and zebrafish, agree that the epicardium is likely to be a heterogeneous cell population formed by different cell subsets, each defined by a specific expression signature, as well as by genes that may represent subset-specific markers [139]. Therefore, it is possible that this disparity in regenerative capacity could stem from differences in the cellular composition of the epicardium due to the presence of subsets of cells that contribute more significantly to cardiac repair [146].

In this context, it has been proposed that the observed heterogeneity in the epicardium may be attributed, at least in part, to the origin and cellular composition of the proepicardium (PE), a heterogeneous cell cluster consisting of endothelial cells (ECs) [147] within a mesenchymal core, covered by an epithelial outer layer, from which most epicardial cells originate. This composition of the proepicardium could influence the multiple cell fates of EPDCs during heart development [148]. Molecular markers with varying spatiotemporal expression in the proepicardium may indicate the presence of distinct cell subtypes with specific roles, dividing the proepicardium into genetically distinguishable compartments by variations in the cellular composition of the epicardium, whether in developmental stages or in the adult heart post-myocardial infarction, with evidence of differences even within the same species [3]. It is generally assumed that the activated epicardium recapitulates the embryonic program in generating mesenchymal progenitor cells, although there may be major molecular differences with respect to their embryonic counterpart [144]. Thus, epicardial EMT reactivation during regeneration doesn’t completely mirror embryonal epicardial EMT, leading to different progenitor states and subpopulations.

In an effort to enhance comprehension of this diversity, Hesse et al. employed scRNA-seq combined with RNA in situ hybridization and lineage tracing to identify various different clusters of epicardial cells in the injured adult mouse heart, 5 days after myocardial infarction (MI), separated in 3 groups: group I cells (expressing Wt1) located in the outermost epicardial layer, group III markers widely distributed across the activated epicardium but predominantly in the inner layers, and group II featuring epithelial clusters (expressing Wt1) as well as inner layer clusters enriched with extracellular matrix-related pathways [149].

Through scRNA-seq analysis and genetic approaches, Xia and colleagues defined two distinct subpopulations (epithelial and mesenchymal) within zebrafish epicardial cells isolated from regenerating hearts [150] alongside a transiently activated epicardial progenitor cell (aEPC) population able to differentiate into mural cells and mesenchymal epicardial cells while providing pro-regenerative factors during regeneration.

Furthermore, the Authors examined the expression of zebrafish cluster markers in the mouse dataset by Hesse and colleagues to compare epicardial composition between zebrafish and mice revealing both similarities and differences in epicardial populations across the two species. They found that mouse clusters I, II, and III corrspond to zebrafish epithelial, aEPC, and mesenchymal/mural subsets, respectively [150]. However different studies have independently verified that, unlike in zebrafish, mouse epithelial epicardial cells do not give rise to mesenchymal epicardial cells in the infarct region, potentially contributing to the limited regenerative capacity of the adult mouse heart [149, 151].

### Lower vertebrates

The epicardium is a key regulator of the regenerative response in the zebrafish heart following injury [139, 152, 153] due to the capacity of epicardial cells to proliferate, undergo EMT and secrete cytokines that stimulate cardiomyocyte cell cycle re-entry [117]. Furthermore, following EMT, these cells adopt an embryonic-like gene expression profile, migrate to the injured region and differentiate into fibroblasts and mural cells that support neovascularization [9, 154]. Growth factors essential for epicardial EMT, such as PDGF, FGF and VEGF, are all ligands for neuropilin (NRP) transmembrane receptors [155–157].

Interestingly, using the zebrafish heart cryoinjury model, Lowe and collaborators demonstrated the significant involvement of NRPs in epicardial EMT and cell motility during zebrafish heart regeneration [158]. Accordingly, zebrafish mutants lacking Nrp1a displayed delayed heart regeneration and impaired revascularization. The authors suggested that these effects were partly attributed to the inhibition of epicardial EMT and the migration of epicardial cells.

In another recent study, employing a zebrafish model of ventricular ablation, the authors demonstrated the critical role of the TGF-β/SMAD3 signaling pathway in heart repair [159]. Importantly, this signaling pathway has been implicated in multiple processes during regeneration, including cardiomyocyte cell cycle progression and EMT. In this study, the involvement of a CM EMT-like response is intriguing since, generally, this phenomenon is associated with the transformation of epicardial cells to a mesenchymal phenotype that is more prone to migration. Surprisingly, this study revealed that proliferating cardiomyocytes near the ablated area exhibited an EMT-like response necessary for their migration into the extracellular matrix located between the myocardium and epicardium to repair the damaged region during cardiac regeneration. Accordingly, SMAD3 inhibition impaired CM migration by weakening this EMT-like response and therefore inhibited ventricular regeneration.

#### Epicardial EMT regulation by different components of the ECM

Several reports have investigated the influence of the matrix on the activation of the epicardium, particularly epicardial EMT, during zebrafish cardiac regeneration. Specifically, in an early report, Missinato and colleagues observed increased expression of a hyaluronic acid (HA) receptor (Hmmr) following ventricular resection in the zebrafish heart. HA, a component of the extracellular matrix (ECM) generated postinjury, accumulates at the wound site, while Hmmr, a cell surface receptor for HA, primarily functions to promote cell motility during wound healing [160]. The authors postulated that the HA pathway could serve important functions in the regenerating heart. Accordingly, suppression of HA production, as well as depletion of Hmmr, hindered cardiac regeneration. Mechanistically, HA (localized within the epicardium) and Hmmr are required for epicardial cell EMT, which facilitates their subsequent migration into the regenerating ventricle to support coronary vasculature formation. It was suggested by the authors that this pathway could also be important for cardiac repair in mammals since, in a rat model of myocardial infarction, both HA and Hmmr were upregulated and localized in the infarcted area within the first few days following damage. Remarkably, these data imply that HA not only acts as a component of the ECM but also potentially functions as a signaling molecule that promotes epicardial EMT [160].

A subset of epicardial cells expressing hapln1, a factor associated with the ECM and required to produce a matrix rich with organized HA at sites of injury, plays a vital role in myocardial repair. According to a recent investigation, these cells interact with regenerating cardiomyocytes, as evidenced by the inhibition of cardiac muscle regeneration after hapln^+^ cell depletion. This hindrance could be attributed to a deficiency in HA deposition by epicardial cells leading to impaired CM proliferation and dedifferentiation during regeneration [161].

Based on the findings of Missinato and colleagues, as well as Sun et al., it is plausible that during zebrafish heart regeneration, hapln^+^ epicardial cells might stimulate the accumulation of HA, which serves a dual purpose: first, by creating a supportive scaffold crucial for CM growth and specialization and, second, by triggering Hmmr-mediated epicardial EMT. This EMT process is responsible for the migration of epicardial cells toward the site of injury, thereby fostering angiogenesis and ultimately facilitating regeneration.

Remarkably, another component of the ECM, the basement membrane-associated proteoglycan agrin, has been previously identified as a key regulator of epicardial EMT.

As mentioned earlier, by inducing epicardial EMT, agrin participates in the differentiation of epicardial cells into different cell types, including fibroblasts and VSMCs, to support coronary vessel formation during mouse cardiac development [104].

It is crucial to emphasize that agrin was previously shown to be involved in heart regeneration in adult mice by exerting pleiotropic effects, such as inhibiting fibrosis, modulating the immune response, promoting angiogenesis and slightly enhancing CM proliferation [162].

A recent study showed that the matrix metalloproteinase MMP-14 is induced in response to heart damage and facilitates regeneration in the zebrafish heart. According to the authors, MMP-14, produced by endothelial cells, likely operates through multiple mechanisms during cardiac regeneration, including degrading scar tissue and promoting angiogenesis and, notably, increasing agrin deposition in the extracellular matrix of neonatal mouse hearts [163].

Therefore, agrin, which is crucial during heart development in supporting epicardial EMT, might also play a significant role in the postnatal heart after injury by stimulating epicardial cells to participate in the formation of new vessels through the EMT process and acquisition of an endothelial phenotype.

#### Novel mediators of epicardial activation

Several hormones affect cardiac regeneration [164, 165]. Oxytocin (OXT) is a neuroendocrine peptide produced by the hypothalamus and released by the posterior pituitary gland. A recent study showed that OXT plays a critical role in proper epicardial development in zebrafish embryos and that OXT signaling seems to be conserved in adult zebrafish [166].

Specifically, this hormone induces a progenitor-like state, increased cell proliferation, EMT, and transcriptional activity in a model of human induced pluripotent stem cell (hiPSC)-derived epicardial cells through the oxytocin receptor (OXTR). Furthermore, its signaling is reactivated following cardiac injury in adult zebrafish, where it is released from the brain into the bloodstream, leading to significant epicardial EMT and migration of Wt1^+^ epicardial progenitor cells into the myocardium to facilitate heart regeneration. The TGF-β pathway, a well-established inducer of EMT, serves as the primary mediator of OXT-induced epicardial activation. Nevertheless, the contribution of OXT-mediated epicardial activation by EMT in zebrafish heart regeneration has not been completely established, as OXT could support a pro-regenerative phenotype by activating not only epicardial cells but also other cell types, possibly through indirect pathways related to the epicardial secretion of pro-regenerative molecules. Therefore, as suggested by the authors, more advanced genetic zebrafish models should be used in the near future to reveal the interactions between epicardial cells and other cells present in the regenerating zebrafish heart [166].

#### New subsets of activated epicardial progenitor cells

The association between the acquisition of a progenitor-like state in the epicardium and zebrafish heart regeneration has also been postulated in another study by Xia and colleagues [150]. Through scRNA-seq analysis and genetic approaches, which include mCherry labeling of the epicardial epithelial layer, the authors detected an average of 26.8% mCherry^+^ cells undergoing EMT to give rise to mesenchymal cells during heart regeneration. These cells represent a transiently activated epicardial progenitor cell (aEPC) population that is essential for heart regeneration, as they differentiate into Pdgfrb^+^ mural cells and mesenchymal epicardial cells and provide pro-regenerative factors during regeneration. These stromal cells (named EpiSCs, i.e., epicardial stromal cells) have also been identified in adult mice and analyzed by scRNA-seq 5 days after myocardial infarction. Many similarities have been found between the two species, but mouse epithelial epicardial cells do not give rise to mesenchymal EpiSCs in the infarcted region, unlike in zebrafish [149].

Notably, aEPCs also differentiate into hapln1a-expressing mesenchymal cells enriched in regenerating epicardial cells present in the wound. Most likely, these cells mediate heart regeneration by orchestrating ECM remodeling.

Epicardial cells are considered not only a source of paracrine signaling and extracellular matrix molecules but also the cells that replenish cardiac muscle following EMT and differentiation in salamanders after cryoinjury [167]. Through the integration of genetic marker-independent lineage-tracing approaches with transcriptional profiling and loss-of-function techniques, Eroglu and collaborators demonstrated that in salamanders, 210 days following heart cryoinjury, there were no signs of damage, and tissue organization was completely restored by epicardial cell differentiation into cardiomyocytes. Specifically, following EMT, EPDCs expressing the tight junction protein CLDN6 migrated into the injury area and differentiated into cardiomyocytes, contributing to tissue regeneration in at least 15% of the regenerated tissue.

### Mammals

#### Neonatal heart

In mammals, regenerative capacity is retained only in the early neonatal period [168, 169]. In neonatal mice, rats and pigs, this capacity is lost one week after birth [168–172]. Evidence from coronary corrosion casts performed at 7 and 21 days after LAD ligation in 1-day-old mice indicates that a vascular response restores perfusion during neonatal heart regeneration [169]. Importantly, the expression of EMT-related genes is upregulated in neonatal mice following cardiac apex resection, indicating the involvement of epicardial EMT in neovascularization [168].

A very recent study demonstrated the regenerative potential of maintaining cardiomyocyte proliferation and tissue regeneration in opossum neonates after apex resection or LAD ligation for at least two weeks after birth, i.e., a longer regenerative time frame than that of neonatal mice [173]. This ability was completely lost by the first month after birth. Nevertheless, it should be noted that marsupials are characterized by postnatal organogenesis, and in this study, cardiomyocyte mitotic activity was detected during postnatal development (for at least 2 weeks after birth), which is a period equivalent to that of neonatal mice at P1. Mechanistically, CM proliferation has emerged as the primary driver of myocardial regeneration, and AMPK signaling appears to be responsible for this process in both opossums and neonatal mice. A direct role for AMPK in EMT has been established [174], and it would be very interesting to investigate the possible involvement of epicardial EMT in marsupial cardiac regeneration.

#### Adult heart

In adult mammals, this dormant fetal growth program is reactivated after MI. Wt1^+^ epicardial cells can recapitulate their embryonic profile during EMT.

The concept of an EMT-like response in cardiomyocytes has been postulated not only in the zebrafish heart [159] but also in the infarcted mouse heart during regeneration [175]. Using the transient ca*Erbb2* CM-specific induction system, MI was induced in adult mice, and caErbb2 was induced after 3 weeks. ERBB2 activation led to cardiac regeneration even after deterioration and scarring of the infarcted heart. Through RNA-seq, significant EMT induction in cardiomyocytes was detected, enabling them to degrade the ECM and migrate into the scar, thus replacing and regenerating the damaged tissue. YAP and ERK were identified as critical mediators of ERBB2 signaling. Notably, this study confirmed the importance of an EMT-like process during cardiac regeneration in the adult mammalian heart.

Among the cytokines and growth factors supporting epicardial EMT, clusterin, a heterodimeric secreted glycoprotein detected in exosomes isolated from the pericardial fluid (PF) of patients with acute MI, was identified [176].

First, we demonstrated that PF affected gene expression in epicardial cells following MI and, specifically, modulated EMT. The impact of the PF was assessed by inducing coronary artery ligation in mice with and without pericardial sac integrity, i.e., in the presence and absence of the PF. Gene expression profiles of epicardial cells isolated from these two groups of mice 3 days following MI revealed that the modulation of EMT-related genes was statistically significant only in the presence of PF [176]. Therefore, we hypothesized that the biological activity of PF could be mediated by exosomes. Accordingly, we isolated exosomes from the PF of control patients and patients with acute myocardial infarction and carried out a proteomic analysis to identify PF-derived soluble factors potentially responsible for epicardial EMT. Among the proteins, clusterin was identified as a heterodimeric secreted glycoprotein already implicated in cancer cell EMT and was found to be highly abundant in PF-MI compared to PF-C. Remarkably, clusterin was also present in plasma-derived exosomes from both control and MI patients, without differences between the two groups, confirming the cardiac origin of this protein under acute conditions. Finally, we showed that treatment with clusterin alone after acute MI *in vivo* promoted epicardial EMT, which enhanced arteriogenesis and protected cardiomyocytes from apoptosis, thus confirming the importance of epicardial EMT in cardiac regeneration [176].

In a recent study, we investigated the role of miRNAs as key regulators of epicardial EMT and their direct targets [177]. In an in vitro model of epicardial mesothelial cell (EMC) EMT, miR-200c-3p was the most prominently suppressed miRNA during TGF-β1-mediated EMT in these cells. FSTL-1 was identified as the direct target of miR-200c-3p. Specifically, epicardial FSTL1 induced EMT in EMCs and boosted their migratory capacity. Importantly, previous studies have recognized this glycoprotein as a protective cardiokine during post-MI cardiac remodeling [178, 179] and regeneration [180] as well as an intrinsic cardiokine that promotes the survival and proliferation of hypoxic MSCs transplanted into the infarcted murine heart [181]. Hence, we hypothesize that, *in vivo*, intrinsic epicardial FSTL1, which is activated following TGF-β1-mediated miR-200c-3p suppression after injury, might induce the EMT of epicardial cells that migrate into the infarcted region and participate in cardiac repair, while secreted epicardial FSTL1 might act as a cardiokine, enhancing cardiomyocyte survival and stimulating angiogenesis.

Interestingly, innovative protocols have recently been implemented in ex vivo settings to investigate the crosstalk between the epicardium and myocardium. Living 3D organotypic epicardial slices from porcine hearts have been recently developed [182]. In particular, slices that included both the epicardium and the myocardium while preserving 3D organization were primed with thymosin b4, resulting in epicardial EMT, enhanced cell motility and differentiation into epicardial-derived mesenchymal cells. This ex vivo model could serve as a valuable tool for investigating the impact of intrinsic and extrinsic factors on triggering the adult epicardium through EMT to unlock its regenerative potential.

ln a very recent study, epicardial EMT was induced in vitro by exposing Wt1^+^ epicardial cells to carboxylic gelatin-methacrylate nanoparticles loaded with ammonium persulfate (NPs/APS) [183]. Ammonium persulfate (AP) is acknowledged as an EMT inducer, and because of its cytotoxicity, it can be safely delivered into cells using nanoparticles (NPs), such as carboxylic gelatin-methacrylate NPs. Subsequently, the internalization of NPs/APS by lysosomes, which are the primary sites of nanoparticle accumulation within cells, promotes the upregulation of genes associated with EMT. *In vivo*, injection of NPs/APS in the infarcted mouse heart enhanced tissue repair and improved cardiac function mainly through epicardial cell EMT induction, which contributed to neovascularization. Mechanistically, NPs/APs stimulate both autophagy and the mTOR pathway within epicardial cells, thus modulating the EMT process. Notably, expanding the EMT process in epicardial cells through exogenous stimulation could represent an effective strategy for ameliorating cardiac repair post-MI.

In addition to signaling mediators, cellular structure changes may influence EMT dynamics. For instance, the primary cilium is a specialized organelle with the ability to sense mechanical and sensory stimuli and transmit them from the cell's surroundings to its interior [184]. Disassembly of the primary cilium during heart development has been demonstrated to induce EMT [185]. In a recent study, primary ciliary disruption was accomplished by preventing intraflagellar transport protein-88 (Ift88), a protein vital to ciliary assembly, using an adenoviral construct encoding a short hairpin RNA against Ift88. Ift88 knockdown promoted EMT in EPDCs in vitro*,* and intramyocardial administration of As-shIft88 in a mouse model of MI improved EMT, myocardial neovascularization and cardiac remodeling. Interestingly, hypoxia-inducible factor 1-alpha (HIF-1α) levels were significantly greater in the Ad-shIft88-treated group than in the control group, and HIF-1α has been identified as a regulator of both EMT and blood vessel development.

The influence of hypoxia signaling on the regulation of cardiac progenitors in the epicardium and subepicardium has been investigated in a previous study. According to this report, the adult epicardium serves as a physiological hypoxic niche housing a metabolically distinct population of glycolytic progenitor cells under the control of HIF-1α [95].

Recently, chronic physiological hypoxia exposure (obtained by placing adult mice in a hypoxic chamber at 10% O2 for 2 weeks) has been shown to activate the epicardium in the adult murine heart [186]. This activation occurs through the induction of EMT, leading to a transition to a progenitor state characterized by multilineage differentiation potential. Specifically, under hypoxic conditions, these Wt1^+^ and Pw1^+^ progenitor cells were able to differentiate into stromal, smooth muscle and endothelial lineages, mirroring the behavior of neonatal epicardial cells.

## Conclusion and outlook

The contribution of the epicardium to cardiac repair and regeneration after damage is now widely acknowledged. Whether it participates as a source of progenitor cells or as a critical signaling hub, it is critical that the epicardium first undergoes the EMT process. Notably, the notion of an EMT-like response has recently been proposed not only in epicardial cells but also in cardiomyocytes both in the zebrafish heart [159] and in the infarcted mouse heart [175], confirming the importance of EMT-like processes during cardiac regeneration.

Recently, novel in vitro models and ex vivo platforms have been established to explore the influence of new signaling pathways and the role of extracellular matrix molecules in activating the adult epicardium via EMT to unleash its regenerative capabilities.

Some key approaches to harness epicardial EMT in pre-clinical settings, that might represent potential therapeutic strategies, are represented, for instance, by the modulation of novel signaling pathways involved in epicardial activation and EMT with small molecules and growth factors. Biodegradabile scaffold or nanoparticles can be used to deliver in a controlled manner bioactive molecules as components of the ECM to provide sustained stimulation of epicardial EMT and ongoing repair processes. microRNAs are known to regulate EMT. Delivering microRNAs that promote regenerative EMT while inhibiting fibrotic EMT can improve cardiac repair outcomes. Another strategy may be represented by cell therapies using specific subset of activated epicardial progenitor cells that are able to stimulate heart regeneration since they differentiate into mesenchymal epicardial cells that provide pro-regenerative factors.

Therefore, the therapeutic modulation of epicardial EMT holds great promise for enhancing cardiac regeneration eventhough there are several challenges to be considered as inducing EMT in a controlled manner to avoid excessive fibrosis or ensuring that these therapies lead to sustained improvement in cardiac function without adverse effects.

Research on epicardial EMT may also be applied in clinical settings through personalized approaches. A cell culture system to efficiently isolate human adult epicardial cells (primary EPDCs) and culture them in their epithelial-like state was established a few years back [187] and more recently inducible proliferative adult human EPDCs (iEPDCs) useful to study human epicardial properties were generated [188]. Further, a protocol for differentiating induced pluripotent stem cells (iPSCs) into epicardial-like cells (iECs) through temporal modulation of canonical Wnt signaling has been developed too [189]. All these cell types can be sourced from patients enabling the evaluation of therapies in vitro and gaining insights into patient-specific responses thus optimizing therapeutic approaches.
